# Incidence and mortality of acute aflatoxicosis: A systematic review

**DOI:** 10.1016/j.envint.2025.109461

**Published:** 2025-05

**Authors:** Tess Goessens, Kokeb Tesfamariam, Patrick Berka Njobeh, Limbikani Matumba, Nyadani Jali-Meleke, Yun Yun Gong, Zdenko Herceg, Chibundu N. Ezekiel, Sarah De Saeger, Carl Lachat, Marthe De Boevre

**Affiliations:** a Centre of Excellence in Mycotoxicology and Public Health, Faculty of Pharmaceutical Sciences, Ghent University, Ottergemsesteenweg 460, 9000 Ghent, Belgium; b Department of Food Technology, Safety and Health, Faculty of Bioscience Engineering, Ghent University, Coupure Links 653, 9000 Ghent, Belgium; c Department of Biotechnology and Food Technology, Faculty of Science, University of Johannesburg, PO Box 17011, Doornfontein 2028, South-Africa; d Food Technology & Nutrition Group, Lilongwe University of Agriculture and Natural Resources, Ntcheu Street Plot 182, Lilongwe, Malawi; e School of Food Science and Nutrition, University of Leeds, Woodhouse Ln, Woodhouse, Leeds LS2 9JT England, United Kingdom; f Epigenomics and Mechanisms, International Agency for Research on Cancer, 150 Cours Albert Thomas, F-69008 Lyon, France; g University of Natural Resources and Life Sciences, Department of Agrobiotechnology (IFA-Tulln), Institute of Bioanalytics and Agro-Metabolomics, Konrad-Lorenz-Strasse 20, Vienna 3430, Tulln an der Donau, Austria

**Keywords:** Aflatoxins, Acute aflatoxicosis, Systematic review, Dietary exposure, Population studies

## Abstract

**Background:**

Aflatoxins are mycotoxins produced by *Aspergillus* fungi in crops intended for food and feed. Acute exposure to high levels of aflatoxin B1, one of the most toxic mycotoxins, can result in severe poisoning, defined as acute aflatoxicosis, which manifests as acute hepatic failure followed by death in severe cases. Currently global burden estimates of acute aflatoxicosis are lacking – in contrast to burden estimates of chronic exposure – making it difficult to implement and prioritize risk management strategies in the prevention and control of aflatoxin exposure.

**Aim:**

This systematic review assessed global evidence on the incidence and mortality of acute aflatoxicosis from 1990 to 2023. While symptomology & disease duration was also examined, it served as a secondary outcome to provide additional clinical context.

**Search Strategy and Eligibility:**

A structured search was conducted in PubMed, Web of Science, Embase, Scopus, INASP and grey literature. Studies were imported into Covidence for review.

**Study Selection and Extraction:**

Two independent reviewers screened and extracted titles, abstracts, and full texts. Eligible studies included all human studies.

**Results:**

From 11,539 references, 9 studies were included. Heterogeneity existed in study design, region, age of the study population and aflatoxin analysis. Number of cases ranged from 1 to 317, with aflatoxin concentrations varying widely, *i.e.* between 10 and 51,100 µg/kg in food, 36 and 209,000 pg/mg albumin in serum, and 19 and 18,521 pg/g in tissue. Only one outbreak provided sufficient data to estimate an attack rate of 8 cases per 100,000. Mortality ranged from 16.2 to 76.5 %, affecting children under 15 and adults over 40 most severely. Common symptoms included vomiting (77–100 %), jaundice (88–100 %), and abdominal pain (8–87 %). The risk of bias was generally low.

**Conclusion:**

This review shows that acute aflatoxicosis remains a significant public health burden, especially among vulnerable groups in African countries, although the variability in studies and lack of standardized reporting make burden estimation difficult, highlighting the need for better warning systems and standardized reporting, despite challenges with infrastructure and resources in affected areas.

## 1 Introduction

Aflatoxins are mycotoxins produced by the *Aspergillus* fungi in certain agricultural crops intended for human consumption and animal feed, such as maize, wheat, millet, sesame seeds, rice, figs, spices, nuts and cocoa, which can be contaminated by fungi during pre- and post-harvest stages (Mahato et al., 2019). Aflatoxin B1 (AFB1), one of the most toxic aflatoxin metabolites, is classified by the International Agency for Research on Cancer (IARC) as a Group 1 carcinogen, directly associated with liver cancer (hepatocellular carcinoma) (IARC, 2012). An estimated five billion people in developing countries, particularly in regions of Asia and Africa, are at risk of uncontrolled exposure to this harmful contaminant due to its high prevalence in food (Strosnider et al., 2006). Sufficient evidence in humans exist for the carcinogenic, immunosuppressive and growth impairing properties of chronic exposure to aflatoxins, as also included in the World Health Organization’s (WHO) first estimates of the global burden of foodborne diseases (Havelaar et al., 2015, WHO, 2016, WHO, 2010), whilst acute exposure to high levels of aflatoxins can result in severe poisoning, defined as acute aflatoxicosis. Acute aflatoxicosis manifests as acute hepatic failure and jaundice, caused by toxic metabolites that lead to hepatic necrosis through mechanisms involving DNA damage, oxidative stress, and lipid peroxidation. Disruption of protein synthesis and immune suppression further contribute to disease severity, and impaired production of clotting factors leads to coagulopathy and bleeding risks (Benkerroum, 2020). As a result, symptoms of acute aflatoxicosis include nausea, vomiting, abdominal pain, fever, diarrhoea, oedema, jaundice, convulsions, intestinal bleedings, and even death in severe cases (Kamala et al., 2018). In the past, the disease often occurred in persons forced by economic circumstances to consume heavily moulded corn containing aflatoxins at concentrations that range between 6 and 16 mg/kg, with an average daily intake of 2 to 6 mg aflatoxin per person (Dhanasekaran et al., 2011).

Currently, global burden estimates of acute aflatoxicosis are lacking (Klingelhöfer et al., 2018), making it difficult to implement and prioritize risk management strategies in the prevention and control of aflatoxin exposure. The WHO together with the Chemical and Toxins Task Force (CTTF) of the Foodborne Disease Burden Epidemiology Reference Group (FERG) for 2021–2025, has developed a workplan that prioritizes various chemicals and toxins, including aflatoxins, for systematic reviews (SRs) to identify the global burden of foodborne hazards and establish an association between the disease associated with the relevant hazard upon exposure via consumption of contaminated food. As such, the specific objective of this study was threefold. First, to provide a rigorous, comprehensive, and balanced evaluation of all relevant literature published between 1 January 1990 and 15 April 2023 on national or (sub-)regional evidence of incidence and mortality of acute aflatoxicosis, updating the existing evidence. Second, to assess if sufficient data exists to estimate the burden of acute aflatoxicosis in collaboration with FERG for 2021–2025 in particular CTTF, on the basis of the above. And third, if relevant, to characterize through scientific literature the health state(s) associated with acute aflatoxicosis including disease symptoms and duration. Health outcomes associated with chronic exposure to aflatoxins are beyond the scope of this review.

## 2 Methods

### 2.1 Problem formulation

#### 2.1.1 Planning

A multidisciplinary review team with expertise in mycotoxicology, human health, nutrition, and systematic review methodologies conducted this study based upon the Handbook for Conducting a Literature-Based Health Assessment Using OHAT Approach for Systematic Review and Evidence Integration (NTP, 2019). An advisory panel of experts provided strategic input during the planning and scoping phases but was not involved in the execution of the work. Funded and commissioned by the WHO, the research was conducted independently, with WHO offering advisory support at the outset and no involvement in the study’s execution or manuscript preparation. Comprehensive details about the review team, advisory board, and sponsor are provided in the Supplementary Materials (Table S1).

#### 2.1.2 Scoping exercise

A scoping exercise was conducted to develop the search string, with a primary focus on estimating disease burden through incidence and mortality measures. The search was tailored accordingly, based on published literature (Darwish et al., 2014, Gong et al., 2016, Nji et al., 2022, Periaca et al., 2014, Williams et al., 2004) and expert consultation processes (TG, CL, MDB, PN, YYG, CNE and LM), resulting in the following keywords: mycotoxin, aflatoxin, fungal toxin, aflatoxicosis, aflatoxin poisoning, aflatoxin toxicity, acute hepatic necrosis, acute liver failure, acute hepatotoxicity and aflatoxin-induced illness. To assess the current need for a *de novo* SR on the health burden of acute aflatoxicosis, detailed electronic search strings were developed in Pubmed, Embase, Cochrane Library, Web of Science Core Collection (WoS) and Scopus, as presented subsequently in Section “2.2.2 Search string”, using the predefined keywords, additionally filtering on ‘review’ or if possible ‘systematic review’. This search yielded a total of 3 articles from PubMed, 4 from Embase, none from the Cochrane Library, 31 from Scopus, and 15 from the Web of Science (WoS). The results are available for open access on the Open Science Framework (OSF) digital platform, specifically in the “Scoping” section, at https://osf.io/zj9kp/. Next, databases commonly used for registrations were searched for existing or ongoing reviews on the topic of mycotoxins and human health using the predefined keywords (PROSPERO, https://www.crd.york.ac.uk/prospero/, (n = 0); OSF Registry, https://osf.io/registries, (n = 0); and Zenodo, https://zenodo.org/, (n = 18)). Of the retrieved articles, only three reviews addressed a similar topic related to mycotoxins. Two of these were not systematically performed, lacking clearly defined Population, Exposure, Comparator,Outcome and Study (PECOS) criteria, search strategies, and quality appraisals. Instead, both studies focused broadly on the toxicological impacts of aflatoxins rather than specifically addressing acute cases (Denning, 1987, Shabeer et al., 2022). The third article comprised a SR focusing on commodity-specific risk assessments for aflatoxins (Bhardwaj et al., 2023).

#### 2.1.3 Setting the research question

To evaluate the incidence and mortality of acute aflatoxicosis in human populations (*i.e.* primary outcomes) and its associated symptomology and disease duration for additional clinical context (*i.e.* secondary outcomes), the following research question was developed based on the obtained evidence within the PECOS framework (Morgan et al., 2018): *Amongst humans of all age and gender (Population), what is the incidence and mortality rate, as well as symptomology and disease duration (Outcome) of acute aflatoxicosis (Exposure), in comparison to either no exposure or a reduced level of exposure (Comparator)?*

##### 2.1.3.1 Population

Studies on (chronic) aflatoxin exposure have primarily focused on populations in Africa and Asia, where aflatoxin contamination of staple crops is widespread. For instance, studies investigating childhood stunting due to aflatoxin exposure have frequently involved African children, particularly in regions like Tanzania and Benin (Gong et al., 2004, Tesfamariam et al., 2022). In studies on liver disease and cancer, the populations often originate from regions in sub-Saharan Africa such as Tanzania and Nigeria as well as parts of Asia including China, and more in areas with high hepatitis B virus (HBV) prevalence, as co-infection with HBV and aflatoxin exposure significantly increases liver cancer risk (Afum et al., 2016, Claeys et al., 2020, Gong et al., 2004). Population age relates to the type of health outcome studied, *e.g.* studies related to stunting involve young children (infants to age 5), while liver and kidney disease, as well as cancer studies primarily focus on adult populations (Afum et al., 2016, Andrews-Trevino et al., 2019, Claeys et al., 2020, Desalegn et al., 2011, Gong et al., 2012, Tesfamariam et al., 2022). In the case of acute aflatoxicosis, it can be expected that young children are more prone because of their larger intake per body weight ratio and their lower capacity to detoxify (Andrews-Trevino et al., 2021). Finally, one-gender studies generally relate to the type of studied outcome, *e.g.* aflatoxin-related adverse pregnancy outcomes and breast cancer in females (Claeys et al., 2020, Tesfamariam et al., 2022).

##### 2.1.3.2 Exposure

Aflatoxins are common in hot, humid regions but can also occur in temperate climates during warm, wet seasons or improper crop storage. *Aspergillus flavus* is widespread and tends to colonize the aerial parts of plants, such as leaves and flowers, producing B-type aflatoxins. *Aspergillus parasiticus*, which favorizes the soil parts of plants, produces both B- and G-type aflatoxins. Other species, such as *A. bombysis*, *A. ochraceoroseus*, *A. nomius*, and *A. pseudotamari*, can also produce aflatoxins but are encountered less frequently. Among the 18 identified types of aflatoxins, the most significant ones are AFB1, aflatoxin B2 (AFB2), aflatoxin G1 (AFG1), aflatoxin G2 (AFG2), aflatoxin M1 (AFM1), and aflatoxin M2 (AFM2). AFM1 and AFM2 are the primary metabolites of AFB1 and AFB2, respectively, in both humans and animals. These metabolites can be found in milk, meat, or eggs from animals that have consumed feed contaminated with AFB1 and AFB2 (Filazi and Sireli, 2013). The most prominent pathway of human exposure to aflatoxins is via acute or chronic consumption of contaminated food. Aflatoxins are widely present in various types of food matrices, including spices, cereals, oils, nuts, fruits, vegetables, milk, meat, and other food products (De Ruyck et al., 2020, Kinyenje et al., 2023, Mahato et al., 2019). Another prominent route of exposure is parental route, that which entails mother-to-child transmission via the placenta or breastfeeding (Memiş and Yalçın, 2019). Less-important routes of aflatoxin exposure include inhalation and dermal uptake, which are often related to occupational exposure (Kemppainen et al., 1988, Liao and Chen, 2005).

##### 2.1.3.3 Comparator

If applicable and measurable, comparators are defined as non-cases, often exposed to lower aflatoxin concentrations, the latter assessed via either food analysis and consumption data, biomonitoring (serum, plasma, whole blood, urine and/or tissue), or physiologically based toxicokinetic modelling. In the case of outbreak reports, the establishment of proper control groups remains challenging due to several factors such as (1) the urgency of the outbreak, requiring immediate response and intervention, and making it difficult to design and implement control groups in real-time, (2) the difficulty in identifying unaffected controls since outbreaks affect specific communities or populations who all have some level of exposure, and (3) the retrospective nature of these studies, forcing researchers to rely on historical data which may not include a well-characterized control group (Fonseca and Haroutune, 1991).

##### 2.1.3.4 Outcome

To date, population studies have examined the effects of chronic aflatoxin exposure on a wide range of different health effects, including stunting (Andrews-Trevino et al., 2019, Gong et al., 2004, Tesfamariam et al., 2022, Voth-Gaeddert et al., 2018), liver disease (Afum et al., 2016, Gong et al., 2012), kidney disease (Desalegn et al., 2011, Díaz de León-Martínez et al., 2019), diabetes (Akash et al., 2021, Alvarez et al., 2022), adverse pregnancy outcomes (Andrews-Trevino et al., 2019, Tesfamariam et al., 2022) and various types of cancer (Claeys et al., 2020). IARC has classified AFB1, AFB2, AFG1, AFG2 and AFM1 as group 1 according to their carcinogenicity to humans, *i.e.* being confirmedly carcinogenic (IARC, 2021). Health effects induced by acute aflatoxin exposure are less common and less studied in humans than chronically-induced health effects (Benkerroum, 2020), since high acute exposure tends to occur only in extreme situations among humans over a short period, such as during food crises, severe contamination incidents, or when food safety measures break down (Kumar et al., 2017).

##### 2.1.3.5 Study design

Acute aflatoxicosis has been studied in animals (Edds, 1973), *in vitro* and *in silico* (Gilbert-Sandoval et al., 2020). The acute, localized, and urgent nature of acute aflatoxicosis outbreaks, combined with the need for immediate public health action, makes an outbreak report the more appropriate format for documenting such cases. As such, this type of reporting is expected to be most common, as opposed to the broader, population-focused approach of cross-sectional studies.

#### 2.1.4 Protocol

To encourage collaborative and transparent practices, the protocol for this SR has been developed based on the Conduct of Systematic Reviews in Toxicology and Environmental Health Research (COSTER) recommendations (Whaley et al., 2020). It has been registered with the International Prospective Register of Systematic Reviews (PROSPERO) under the ID CRD42023445372 (https://www.crd.york.ac.uk/prospero). Furthermore, both the protocol and SR have been documented as an open-access record on the OSF platform (https://osf.io/zj9kp/) to promote transparency throughout the study’s period.

##### 2.1.4.1 Deviations from the protocol

In the protocol, the terms “health states” and “disease duration” were initially described as “contributing factors,” but this wording has been revised to explicitly refer to symptomatology and disease duration. Additionally, to address the challenge of scattered region-specific data in Africa, the African Union was incorporated as an additional source of grey literature, and secondary literature was accepted as a study type.

### 2.2 Search strategy

#### 2.2.1 Information sources

##### 2.2.1.1 Bibliographic databases

The following databases were selected and a customized search string was constructed for each: Embase, Medline (PubMed), Web of Science and Scopus. Additionally, the International Network for Advancing Science and Policy (INASP) Journals Online project was included, which is an online platform for peer-reviewed journals from developing countries. This platform was expected to play a greater role in acute aflatoxicosis due to less stringent regulations in those regions (INASP, 1998).

##### 2.2.1.2 Grey literature

Several scientific websites were searched, including Google Scholar, OALster and The WHO’s Institutional Repository for Information Sharing (IRIS). Additionally, the African Union (AU) was consulted, being a continental organization comprising 55 African countries, aimed at promoting unity, peace, and development across the continent. The ongoing collaboration between members of the review team and AU members was of the highest value due to their direct experience with the issue, potentially providing region-specific data and insights that enhance the accuracy and relevance of the review.

##### 2.2.1.3 Bibliographic references

Bibliographic references in the papers selected for final analysis were additionally screened to ensure retrieval of all relevant human studies (= citation searching).

#### 2.2.2 Search string

Detailed search strings were developed in EMBASE, PubMed, Web of Science, and SCOPUS based on the keywords identified during the scoping phase. These strings were rigorously reviewed through consultations between the review team and the advisory panel. Two benchmark articles were used to refine and validate the search strategy across all bibliographic databases: one on the 2004 acute aflatoxicosis outbreak in Kenya (Nyikal et al., 2004) and another on the 2016 outbreak in Tanzania (Kamala et al., 2018). The final search strings for each database are provided in Supplementary Material (Table S2). Additionally, the same keywords were used to search all publications within eight journal platforms on the INASP website, as well as the IRIS (WHO), OAIster, and Google Scholar platforms.

### 2.3 Study selection

#### 2.3.1 Inclusion and exclusion criteria

A PECOS approach for defining eligibility was used to develop criteria for this SR (Goessens et al., 2024). Briefly, humans studies, namely cohort studies, (non–) randomized controlled trials, (non–)case-control studies, cross-sectional studies, case and outbreak reports, that contain primary research data on the number of cases, incidence, number of deaths and mortality rate (primary outcomes), as well as the symptomology and disease duration (secondary outcomes) resulting from dietary, as well as non-dietary acute aflatoxin exposure, were included. Populations of all age, gender, health status or life stage at the outcome assessment were considered. All published primary research articles, except for review studies, expert opinions, conference abstracts, presentations, posters and overviewing book chapters were included. These sources were omitted due to their reliance on secondary data, which lacks the original, detailed evidence needed to accurately assess the incidence of acute aflatoxicosis. There were no restrictions on written language. Although animal and mechanistic studies (*i.e. in vivo*, *ex vivo*, *in vitro*) offer valuable insights into mechanisms of action, their differences from human physiology limit their translational relevance. By excluding such studies, the evidence-based conclusions remain robust and specifically focused on human acute aflatoxicosis (Van Norman, 2019). Finally, by searching data from 1990 onwards, the inclusion of more reliable, relevant, and methodologically consistent studies, which can lead to stronger conclusions and actionable recommendations for public health interventions, was ensured (Alameri et al., 2023).

All inclusion and exclusion criteria of this SR according to the PECOS for eligibility framework, are presented in Table 1.

**Table 1:** Inclusion and exclusion criteria of the SR according to the PECOS for eligibility framework.

**Inclusion criteria**	**Exclusion criteria**
***Population***	
Human − No restrictions on age, gender, health status or life stage at the exposure or outcome assessment. Only whole organisms are considered.	Animals and human organs, tissues, cell lines and cellular components.
***Exposure***	
Acute dietary or non-dietary (*e.g.* inhalation, dermal uptake relating to occupational exposure) exposure to aflatoxins, either measured (*e.g.* environmental and consumption data, or biomonitoring studies) or modelled (*e.g.* physiologically based pharmacokinetic modelling).	Other mycotoxins.
***Comparator***	
Human − comparators are defined as non-cases, often exposed to lower aflatoxin concentrations, the latter assessed via either food analysis, consumption data, biomonitoring (serum, plasma, whole blood, urine and/or tissue), or physiologically based toxicokinetic modelling.	Animals and human organs, tissues, cell lines and cellular components.
***Outcome***	
Acute aflatoxicosis, divided into primary outcomes (*i.e.* number of cases, incidence, number of deaths and mortality rate) and secondary outcomes (*i.e.* symptomology and disease duration), and either diagnosed through clinical signs^A^, laboratory tests^B^ and/or exposure history^C^.	Health outcomes related to chronic aflatoxin exposure (*i.e.* cancer, liver cirrhosis, growth stunting, kidney disease, diabetes, adverse pregnancy outcomes).
***Study design***	
Cohort studies, cross-sectional studies, (non–)randomized controlled trials, (non–)case-control studies, case reports and outbreak data, published between 1 January 1990 and 15 April 2023.	Review studies, expert’s opinions, conference abstracts, presentations, posters and overviewing book chapters.

A = Abdominal pain, vomiting, diarrhoea, fever, jaundice (yellowing of the skin and eyes), and/or liver failure, assessed by health care facility/professional.

B = liver function tests, coagulation profile, complete blood count, imaging, aflatoxin detection in biological matrices.

C = history of aflatoxin-contaminated food consumption assessed through food analysis and/or biological sample analysis.

#### 2.3.2 Selection process

All references retrieved from Pubmed, Embase, Web Of Science, Scopus, INASP, grey literature sources and citation searching, were uploaded into Covidence (www.covidence.org), either as a PubMed or RIS text format. Within Covidence, all duplicates were automatically removed, and *Screening on Title and Abstract* was performed independently by two different reviewers (TG and NJ). Only studies that met the set criteria were considered for *Full Text Review*. If there was any disagreement, it was resolved by a third reviewer using a two-third majority decision (CL). The approach to addressing missing data involved obtaining full articles either through a payment service or by leveraging established communication channels with the AU. Finally, data from the eligible studies were extracted in a data extraction template, defined and piloted by Sciensano, Belgium, commissioned by WHO to support FERG’s activities, taking into account the primary research question and PECOS elements, to cover the following information: general details about the input source, specifics about the study design, location, population & time, demographic information of both the cases and (if any) controls, sample size, variables concerning the output estimates of acute aflatoxicosis, *i.e.* number of cases and controls, incidence, number of deaths, mortality, and the type and duration of symptoms. To ensure the repeatability of data extraction and minimize inconsistencies, each article was independently extracted twice by two reviewers (TG and NJ). The data extraction template related to this SR can be consulted via https://osf.io/zj9kp/.

### 2.4 Evidence synthesis

Based upon the expected heterogeneity identified during scoping in terms of outcome measure, study design, population and aflatoxin (matrix) assessment (Tesfamariam et al., 2019), a narrative approach was deemed appropriate for evidence synthesis, following the Synthesis Without Meta-analysis (SWiM) guideline. Following the SWiM guideline, we clustered studies based on measured outcomes (incidence, mortality, symptomology and disease duration) to facilitate meaningful comparisons across age groups and affecting aflatoxin concentrations levels, and minimize bias in interpretation (Campbell et al., 2020). Firstly, a detailed summary of the included studies and their characteristics, *i.e.* study design, study period, study location, population gender ratio and age, and details about the aflatoxin analysis, were provided in a tabular format and narratively discussed (“*3.2 Study characteristics*”). Next, the included studies were clustered according to primary (“*3.4.1 Incidence*” and “*3.4.2 Mortality*”) and secondary outcomes (“*3.4.3 Symptoms*” and “*3.4.4 Disease duration*”), each again presented in a table format and elaborated upon through narrative discussion. Within each cluster, additional criteria were added for narrative analysis. As such, next to the number of cases, the “*Incidence*” discussion included the age distribution and sample size of the studied population to evaluate the incidence by age category and determine the disease-attack rate, respectively. Additionally, to explore the concentrations at which acute aflatoxicosis occurs, measured aflatoxin levels were included. Similarly, within the “*Mortality*” discussion, age distribution and number of cases and fatalities were presented to evaluate potential age predispositions and calculate the case fatality rate, respectively, next to the measured aflatoxin levels able to induce death. Within the “Symptoms” paragraph, symptomology of cases were discussed alongside the data collection methods to give an insight into the reliability of the recorded features. Finally, the disease duration (“*Duration of Disease*”) was presented alongside the number of cases for which this was recorded (as this could differ from the absolute number of cases), as well as the types of symptoms/pathology/disease/survival for which this was recorded.

### 2.5 Quality of evidence

#### 2.5.1 Risk of bias for individual studies

To assess the internal validity of the included studies, a risk of bias (RoB) assessment was performed using the OHAT RoB tool (https://ntp.niehs.nih.gov/go/riskbias). This tool was chosen for its flexibility and applicability to a range of study types, including cross-sectional studies and case or outbreak reports. While not all criteria within the tool are directly relevant to every study design, its selective application ensures a systematic and robust assessment of evidence quality. Given the absence of a universally recognized tool specifically designed for outbreak reports, the review team reached a consensus to adopt the OHAT tool, a decision formalized in the study protocol. In general, the assessment can be conducted via 11 questions depending on the type of study design, being categorized under six potential sources of bias: selection, confounding, performance, attrition/exclusion, detection, and selective reporting. For each RoB question, reviewers must choose between low and high RoB options. Tailored to this SR, appraisal was performed by means of 4 questions, appropriate for the included study types (*i.e.* cross-sectional studies, case reports and outbreak data):

1. Did the study design or analysis account for important confounding and modifying variables?
2. Can we be confident in the exposure characterization?
3. Can we be confident in the outcome measure?
4. Were all measured outcomes reported?

To assess the quality and reliability of included studies, predefined criteria were applied to answer the four questions pertaining to study design rigor (question 1), exposure measurement (question 2), diagnosis confirmation (question 3), and outcome documentation (question 4). Each question was responded with ‘Definitely low,’ ‘Probably low,’ ‘Probably high,’ or ‘Definitely high’ based on specific benchmarks, as detailed within Table S3 of Supplementary. In brief, study design was evaluated by matching groups (Truong et al., 2022), reliable biological measurements (Mahfuz et al., 2020), and accounting for confounders (Kimani, 2022). Exposure measurement depended on the type and reliability of the AF assessment (*i.e.* via biological samples, food or with little or no information on the assessment) (Mahfuz et al., 2020). Diagnosis confirmation was classified based on hospital-based verification, trained personnel, or self-reported questionnaires. Finally, outcome documentation was assessed through hospital records, medical records, or (in-)direct proof of non-documentation.

The assessment was conducted by two reviewers (TG and MDB) and reviewed by a third reviewer (CL) to resolve potential conflicts (two-third majority approach). All authors (TG, KT, CL, MDB, SDS, PN, LM, NJ, CNE, YYG and ZH) reviewed the final assessment, with the opportunity to raise any concerns to ensure a final decision based on majority consensus. After responding to each question, a justification was provided in a separate text field, outlining the study design, confounding, or observations that support the decision, along with any concerns raised during the final decision-making process. All this information was recorded in Microsoft Excel sheets, which can be consulted at https://osf.io/zj9kp/. Based upon the results from each question, an overall low (3 out of 4 questions answered with low RoB), uncertain (equal amount of questions answered with low and high RoB) or high (3 out of 4 questions answered with high RoB) RoB was attributed to each individual study (NTP, 2019). Results were conveyed both narratively and through visual representations. This included a written summary, as well as a traffic light plot, which organized the evaluation for each study across various domains, and a weighted bar plot, which shows the proportion of RoB judgment separately for each domain, using the Robvis visualization tool (Robvis Visualisation Tool, 2022). Finally, in accordance with the OHAT handbook guidelines, and given that each reported case of acute aflatoxicosis contributes valuable data on incidence, mortality, disease duration, and symptoms, studies with high RoB were not excluded from the final synthesis (NTP, 2019).

#### 2.5.2 Certainty of body of evidence

To assess the overall certainty of evidence (CoE), the Grading of Recommendations Assessment, Development and Evaluation (GRADE) approach is generally recommended. In the GRADE approach, randomized controlled trials receive a starting rating of “high,” while observational (non-randomized) studies begin at “low.” Certainty can then be downgraded due to factors like inconsistency (*e.g.* difference in effects, measurement of outcomes or RoB between studies), indirectness (*e.g.* comparison of different populations or exposures), imprecision (*e.g.* small sample sizes), or publication bias (*e.g.* file-drawering effect). It can be upgraded for factors such as a high magnitude of effect (>50 % risk reduction or odds ratio between 2 and 10), clear dose–response gradients, or minimal plausible confounding (NTP, 2019). The CoE assessment process was conducted in duplicate using the same quality controls as previously (see lines 380–386).

## 3 Results

All results, including the included eligible studies, complete data extraction as well as quality assessment, can be consulted upon request via https://osf.io/zj9kp/.

### 3.1 Study selection and flow diagram

**Fig. 1:**
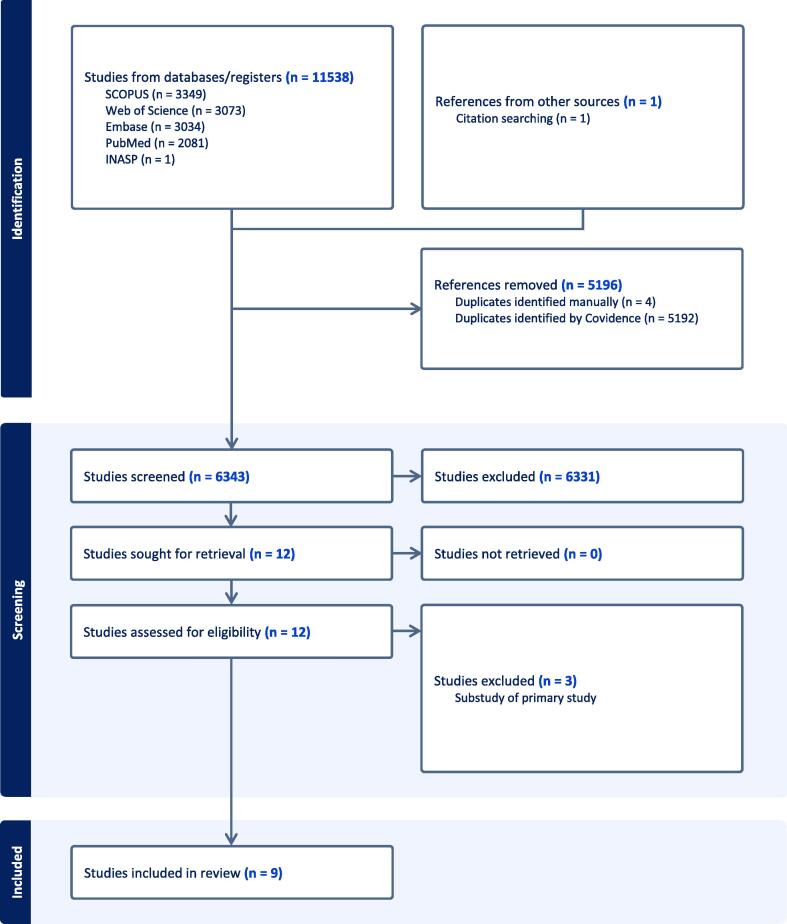
PRISMA flowchart adapted to the current systematic review (Page et al., 2021).

A total of 11,539 references were retrieved from Pubmed (n = 2,081), Embase (n = 3,304), Web Of Science Core Collection (n = 3,073), SCOPUS (n = 3,349), INASP (n = 1), grey literature sources (n = 0) and citation searching (n = 1), and uploaded into Covidence (https://www.covidence.org). All duplicates were automatically removed (n = 5,196), and Screening of Title and Abstract was performed on 6,343 articles, independently, in parallel, by two different reviewers (TG and NJ). Only studies that met the set criteria were considered for Full Text Review (n = 12). No disagreements needed to be resolved by a third reviewer. Of the retained 12 studies, 3 studies, dealing with a subset of data originating from the primary study, were excluded, to result in a final total of 9 studies (Jolly et al., 2007, Kamala et al., 2018, Kinyenje et al., 2023, Mwanda et al., 2005, Nyikal et al., 2004, Ombui et al., 2001, Perduri and Gobba, 2009, Samuel, 2009; Tzee‐Cheng et al., 1991). For one study, no full text was available and extracted information resulted from the abstract only (Mwanda et al., 2005). A detailed overview of the selection process is presented in the PRISMA flowchart in Fig. 1.

### 3.2 Study characteristics

A detailed overview of the different studies and their characteristics is given in Table 2.

**Table 2:** Characteristics of the eligible studies included in this systematic review, alphabetically ordered.

**Source**	**Study type**	**Study period**	**Location**	**Sex**	**Age in years**		**Aflatoxin analysis^LOD in ng/ml (serum) or µg/kg (food)^**
					**Range**	**Mean**	
Jolly, P. 2007	Cross-sectional study	2007	Ghana^A^	Both	19–86	40.8	AFB1 albumin adduct in plasma by ELISA^not specified^
Kamala, A. 2018	Outbreak report	2016	United Republic of Tanzania^B^	Both	0–5	−	AFB1 albumin adduct in serum by ELISA^0.05^ + food from affected households by LC-MS/MS^0.14-0.53^
Kinyenje, E. 2023	Outbreak report	2009	United Republic of Tanzania^C^	Both	−	−	AFB1 lysine adduct in serum – technique not specified^0.03^
Mwanda, O. 2005	Case report	2005	Central African Republic	Male	17	17	Aflatoxins in serum^not specified^
Nyikal, J. 2004	Outbreak report	2004	Kenya^D^	Both	22.1 % <5 years; 29.2 % 5–14 years; 48.7 % >15 years	−	Aflatoxins in food from affected areas^not specified^
Ombui, J. 2001	Cross-sectional study	1970–1993	Kenya	Both	−	−	−
Perduri, R. 2009	Case report	2004–2007	Italy	Female	38	38	Aflatoxins in feed from affected area^not specified^
Samuel, N. 2009	Case report	2008	Israel	Male	28	28	Aflatoxins in consumed food^not specified^
Tzee-Cheng, C. 1991	Outbreak report	1988	Malaysia^E^	Both	3–49	−	Aflatoxins in various tissue extracts by HPLC-ELISA and ICH^not specified^

Note: − = not reported; LOD = Limit of Detection; ELISA = Enzyme-Linked Immuno Sorbent Assay; ICH = Immunohistochemistry;

A = Four villages (Dromankuma, Nkwanta, Hiawoanwu and Kasei) in the Ejura Sekyeredumase district, Ashanti region of Ghana;

B = Dodoma and Manyara regions;

C = Kiteto, Chemba and Kondoa districts;

D = Makueni, Kitui, Machakos, Thika, Embu, Mbeere and Mwingi districts, as well as patients in Kenyatta National Hospital, Nairobi;

E = Perak State;

Four studies were outbreak reports, 3 studies were case reports, and 2 studies were cross-sectional studies. Most studies reported on acute aflatoxicosis occurring between 2004 and 2016, with the exception of 2 studies, reporting on acute aflatoxicosis between 1970 and 1993 (but published much later) (Ombui et al., 2001), as well as in 1998 (Tzee‐Cheng et al., 1991), respectively.

Most of the studies were conducted in Africa (n = 6): 2 in Kenya, 2 in the United Republic of Tanzania, 1 in the Central African Republic and 1 in Ghana. Additionally, 1 study was conducted in Asia (Malaysia), 1 in the Middle-East (Israel) and 1 in Europe (Italy). Except for one study in Kenya (Ombui et al. 2001), all studies were conducted on a sub-national level, reporting the cases of acute aflatoxicosis in districts (Jolly et al., 2007, Kinyenje et al., 2023, Nyikal et al., 2004), regions (Kamala et al., 2018), states (Tzee‐Cheng et al., 1991) and/or hospitals (Kinyenje et al., 2023, Mwanda et al., 2005, Perduri and Gobba, 2009, Samuel, 2009).

All studies were conducted including both sexes except for the case reports. For two studies (Kinyenje et al., 2023, Ombui et al., 2001), no information on age was reported. All other studies included populations aged between 0 and 86 years, with only one study reporting an average age of 41 years (Jolly et al., 2007). In the outbreak report of Nyikal et al. (2004), more than 50 % of patients were less than 15 years of age (Nyikal et al., 2004). The age of individuals included in the case reports were 17 (Mwanda et al., 2005), 28 (Samuel, 2009) and 38 years (Perduri and Gobba, 2009).

Finally, different exposure matrices were examined for the diagnosis of acute aflatoxicosis. The majority of the studies examined aflatoxins (either as AFB1 albumin adduct, AFB1 lysin adduct, or not specified which form and referred to as ‘aflatoxins’) in plasma or serum (n = 4), whilst 3 studies examined aflatoxins in food or animal feed, either from the affected areas (n = 2) (Nyikal et al., 2004, Perduri and Gobba, 2009), or from directly consumed food samples (n = 1) (Samuel, 2009). One study examined AFB1 content in post-mortem tissue extracts (*i.e.* liver, kidney, spleen, lung, heart and brain) (Tzee‐Cheng et al., 1991), and for 1 study (Ombui et al., 2001), no information on aflatoxin analysis was reported. Only 3 studies referenced the used analytical technique, which was primarily based on immunoassays, *i.e.* enzyme-linked immunosorbent assays (ELISA) (Jolly et al., 2007, Kamala et al., 2018; Tzee‐Cheng et al., 1991) and immunohistochemistry (ICH) (Tzee‐Cheng et al., 1991), however one study also analyzed food (maize) samples by means of LC-MS/MS (Kamala et al., 2018). Finally, only 2 studies reported the limit of detection (LOD) of the analytical methodology for aflatoxin quantification, highlighting a tenfold variation in sensitivity based on the analyzed matrix: an LOD of 0.03 ng/mL for AFB1 lysine adduct in serum (technique not specified) (Kinyenje et al., 2023) compared to an LOD of 0.53 (AFB1), 0.15 (AFB2), 0.24 (AFG1) and 0.14 µg/kg (AFG2) in food (maize) and an LOD of 0.05 ng/mL for AFB1 albumin adduct in serum via ELISA (Kamala et al., 2018).

### 3.3 Evidence synthesis

#### 3.3.1 Incidence

A detailed overview of the reported cases of acute aflatoxicosis is given in Table 4, covering either single cases (n = 1), outbreak cases (n between 17 and 317) or cross-sectional studies (n between 24 and 27). The studied population sample size was reported in only one study, *i.e.* the outbreak report of acute aflatoxicosis in the United Republic of Tanzania, covering 3 local districts (Kinyenje et al. 2023), resulting in an attack rate of 8 cases per 100,000. This disease-attack rate was calculated based on the following formula: (number of new cases/population at risk) x 100,000. In the outbreak report of Nyikal et al. 2004, only the summed population for Makueni and Kitui districts were stated, excluding the population counts for Machakos, Thika, Embu, Mbeere and Mwingi districts, as well as Nairobi (Nyikal et al., 2004). For all other studies, population sample sizes were missing. Case-reported aflatoxin concentrations ranged between 10 and 51,100 µg/kg in food, 36 and 209,000 pg/mg albumin in serum, and 19 and 18,521 pg/g in tissue (in respectively heart and kidney). Concentrations found in food are slightly lower, equal to, or often exceed the maximum levels set by the European Commission for the combined total of AFB1, AFB2, AFG1, and AFG2 in foodstuffs such as peanuts (4 µg/kg), dried fruits (4 µg/kg), cereal products (4 µg/kg), almonds (10 µg/kg), and maize and rice (10 µg/kg) (European Commission, 2023). Finally, one study reported consumption of meat resulting from home-raised, highly exposed animals (based on animal feed with AFB1 levels up to 25 μg/kg, exceeding the maximum limit set by the European Commission for complete feed, *i.e.* 10 µg/kg), though there is no aflatoxin information from the consumed food (Perduri and Gobba, 2009, The European Parliament and Council of the European Union, 2002).

**Table 4:** Cases of acute aflatoxicosis reported per study.

**Source**	**Age in years**	**Cases**	**Population sample size**	**Aflatoxin concentration**
Jolly, P., 2007	19–86	27^A^	−	≥ 250 pg/mg albumin in serum
Kamala, A. 2018	0- >40	68	−	10–51,100 µg/kg in food^C^ 36–32,800 pg/mg albumin in serum^C^
Kinyenje, E. 2023	−	62	807,643 (attack rate of 8 per 100,000)	Mean of 209 ng/mg albumin in serum^D^
Mwanda, O. 2005	17	1	−	−^E^
Nyikal, J. 2004	22.1 % <5 years; 29.2 % 5–14 years; 48.7 % >15 years	317	1,286,967^B^	20–8,000 µg/kg in food^F^
Ombui, J. 2001	−	24	−	−
Perduri, R. 2009	38	1	−	25 µg/kg feed^G^
Samuel, N. 2009	28	1	−	19.6 µg/kg in food
Tzee-Cheng, C. 1991	3–49	17	−	AFB1: 38–3,465 pg/g; AFB2: 19–631 pg/g; AFM1: 20–18,521 pg/g; AFM2: 348––5,244 pg/g; AFG1: 9,116 pg/g Aflatoxicol: 27 pg/g in tissue^H^

Note: − = not reported, A = these are persons with symptoms of acute aflatoxicosis (painful vomiting) & high AFB1 levels, however, additionally 9 and 13 persons were reported with other aflatoxicosis symptoms but it is unclear from the article if these originate from the same 27 persons, B = this is the summed population for Makueni and Kitui districts as stated in the article – population counts for Machakos, Thika, Embu, Mbeere and Mwingi districts, as well as Nairobi are missing, C = range presented for AFB1, AFB2, AFG1 and AFG2 together, D = analyzed for 45 cases, E = mention of elevated levels in abstract − no full text available, F = analyzed for 15 out of 31 household food samples, G = consumption of meat resulting from high-exposed animals (animal feed with AFB1 levels up to 25 μg/kg), H = liver, kidney, spleen, lung, heart and/or brain of 13 deceased children.

##### 3.3.1.1 Mortality

Mortality was reported in a health facility or hospital setting by 4 outbreak reports, resulting in a case fatality rate of 29.4 % (Kamala et al., 2018), 16.2 % (Kinyenje et al., 2023), 39.4 % (Nyikal et al., 2004) and 76.5 % (Tzee‐Cheng et al., 1991). In the study of Kamala et al. (2018), highest mortality rates were found for age groups > 40 (53.9 %, CI = [25–81 %]) and ≤ 15 years of age (23.5 %, CI = [7–50 %] − 33.3 %, CI = [15–57 %]), however taking into account widely overlapping confidence intervals which can have an influence on the results (see Table 5). Mortality rates in the study of Tzee-Cheng et al. (1991) should be interpreted with caution, as co-intoxication with excessive levels of boric acid may have influenced the severity of the acute aflatoxicosis (Tzee‐Cheng et al., 1991). Furthermore, none of the 3 case reports of acute aflatoxicosis resulted in any fatality (Mwanda et al., 2005, Perduri and Gobba, 2009, Samuel, 2009), and no mortality data was provided in either of the two cross-sectional studies (Jolly et al., 2007, Ombui et al., 2001).

**Table 5:** Mortality cases and rate associated with acute aflatoxicosis.

**Source**	**Age in years**	**Cases of acute aflatoxicosis**	**Deaths**	**Case Fatality Rate**	**AFB1 concentration**
Kamala, A. 2018	0- >40	68	20	29.4 %	10–51,100 µg/kg in food^A^ 36–32,800 pg/mg albumin in serum^A^
*Kamala, A. 2018*	0–5	17	4	23.5 %^CI=[7-50%]^	−C
*Kamala, A. 2018*	6–15	21	7	33.3 %^CI=[15-57%]^	−B
*Kamala, A. 2018*	16–30	11	2	18.2 %^CI=[2-52%]^	−B
*Kamala, A* *2018*	31–40	6	0	0 %^CI=[0-46%]^	−B
*Kamala, A.* *2018*	>40	13	7	53.9 %^CI=[25-81%]^	−B
Kinyenje, E. 2023	−	62	10	16.2 %	Mean of 209 ng/mg albumin in serum^C^
Nyikal, J. 2004	22.1 % <5 years; 29.2 % 5–14 years; 48.7 % >15 years	317	125	39.4 %	20–8,000 µg/kg in food^D^
Tzee-Cheng, C. 1991	3–49	17	13	76.5 %	AFB1: 38–3,465 pg/g; AFB2: 19–631 pg/g; AFM1: 20–18,521 pg/g; AFM2: 348––5,244 pg/g; AFG2: 9,116 pg/g Aflatoxicol: 27 pg/g in tissue^E^

Note: CI = Confidence Interval;

A = range presented for AFB1, AFB2, AFG1 and AFG2 together,

B = not reported per age group,

C = analyzed for 45 cases,

D = analyzed for 15 out of 31 household food samples,

E = liver, kidney, spleen, lung, heart and/or brain of deceased children.

##### 3.3.1.2 Symptoms

A detailed list of reported symptoms resulting from acute aflatoxicosis is presented in Table 6. Except for 1 study performed via on-site surveys (*i.e.* Jolly et al. 2007), symptomology data were collected in a hospital setting, either by direct investigation or by hospital document reviews. Excluding the case reports, three studies reported sufficient data to estimate the aflatoxin-induced symptomology rate (Kamala et al., 2018, Kinyenje et al., 2023; Tzee‐Cheng et al., 1991). Overall, major reported symptoms include vomiting (between 77 and 100 %), jaundice/yellow mouth (between 88 and 100 %) and abdominal swelling/pain (between 8 and 87 %). Other symptoms include fever (between 46 and 65 %), diarrhoea (between 31 and 50 %), ascites (50 %), scrotum swelling (8 %), rectal bleeding, easy bruisability, constipation, dyspnea, hypothermia, shock, fits (85 %) and coma (32 %). In the study by Jolly et al. (2007), elevated aflatoxin levels (≥250 pg/mg albumin in serum) were associated with a higher risk of painful vomiting, swollen stomach, and yellow mouth, with corresponding odds ratios of 2.2, 3.3, and 5.1, respectively (Jolly et al., 2007). The oliguria symptoms reported in cases from the study of Tzee-Cheng et al. (1991) were not associated with the aflatoxin intoxication but attributed to the co-intoxication with boric acid, which is a kidney toxin that induces metabolic acidosis and acute kidney failure, in contrast to aflatoxins being known hepatotoxins (Tzee‐Cheng et al., 1991).

**Table 6:** Reported symptoms & disease duration associated with acute aflatoxicosis.

**Source**	**Symptoms**	**Duration**	**Data collection**
Jolly, P. 2007 A	Vomiting: 27 (OR = 2.2) Jaundice/yellow mouth: 9 (OR = 5.1) Abdominal swelling/swollen stomach: 13 (OR = 3.3)	−	Surveys on-site
Kinyenje, E. 2023	Jaundice: 62 (100 %) Vomiting: 48 (77 %) Abdominal swelling: 40 (65 %) Fever: 40 (65 %) Scrotum swelling: 5 (8 %)	1–34 days (median: 5 days)	Hospital document reviews + survey on-site
Kamala, A. 2016	Jaundice: 60 (88 %) Abdominal pain: 59 (87 %) Vomiting: 56 (82 %) Diarrhoea; 34 (50 %) Ascites: 32 (50 %)	−	Health care facility document reviews + epidemiological investigation on-site
Mwanda, O. 2005	Vomiting Abdominal distention Tenderness Rectal bleeding Easy bruisability	−	Hospitalized case
Perduri, R. 2009	Nausea Abdominal swelling Constipation Diarrhoea	33 months^C^	Hospitalized case
Samuel, N. 2009	Nausea Vomiting Tenderness/Abdominal pain Dyspnea Hypothermia Shock	48 days	Hospitalized case
Tzee-Cheng, C. 1991^B^	Vomiting: 13 (100 %) Fits: 11 (85 %) Fever: 6 (46 %) Coma: 4 (31 %) Diarrhoea: 4 (31 %) Abdominal pain: 1 (8 %) Jaundice: 13 (100 %)	2–6 days (median: 4.4 days)^D^	Hospitalized cases

Note:

A = total number of cases is unclear from the article,

B = Symptoms were only presented for the 13 persons that died out of the 17 cases,

C = repetitive episodes of acute aflatoxicosis endured due to repetitive exposure, without clear specification of duration of each episode,

D = survival time.

##### 3.3.1.3 Duration of disease

The duration of symptoms and/or pathology associated with acute aflatoxicosis was reported in 4 studies (Table 6), and is presented for all symptoms and/or pathologies together. Duration ranged between 1 and 48 days, with medians between 4.4 (Tzee‐Cheng et al., 1991) and 5 days (Kinyenje et al., 2023), except for a case report in which repetitive episodes have been recorded over the course of 33 months suggested to be related to recurrent intake of highly contaminated meat (Perduri and Gobba, 2009). Aside from the study by Kamala et al. (2018) (Kamala et al., 2018), which collected weekly reports from discharged patients, none of the other studies implemented a well-defined follow-up plan.

### 3.4 Quality of evidence

#### 3.4.1 Risk of bias for individual studies

**Fig. 2:**
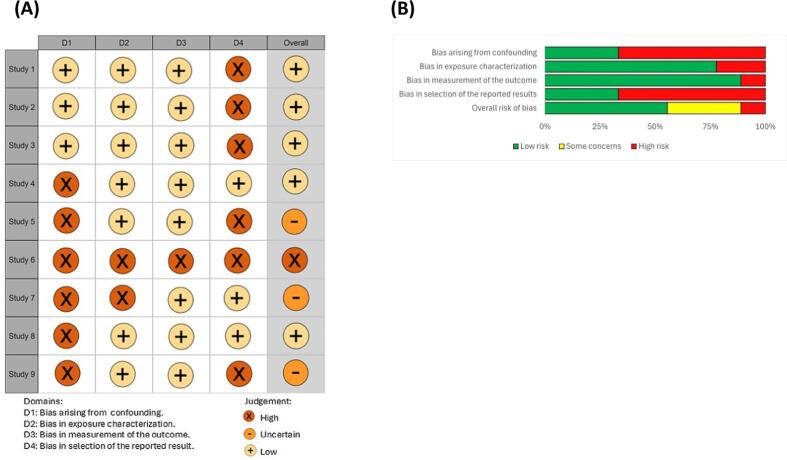
(A) Traffic light plot of the RoB assessment created using robvis and the colourblind palette, (B) RoB summary plot created using robvis and the standard Cochrane palette (Robvis Visualisation Tool, 2022). Note: Study 1 = Jolly et al., 2007; Study 2 = Kamala et al., 2018; Study 3 = Kinyenje et al., 2023; Study 4 = Mwanda et al., 2005; Study 5 = Nyikal et al., 2004; Study 6 = Ombui et al. 2001; Study 7 = Perduri and Gobba, 2009; Study 8 = Samuel, 2009; Study 9 = Tzee-Cheng et al., 1991.

A detailed overview of the RoB assessment of the different studies is given in Fig. 2A and B including the rationale of decisions in Table S3 of Supplementary Materials.

Based upon appropriate matching, aflatoxin analysis method and information on co-exposure or other influencing factors, 6 out of 9 included studies scored poorly for confounding and consideration of modifying variables (**Question 1**), *i.e.* no matched controls, no information on analytical methodology, not taking into account influencing factors, resulting in a definitely (Ombui et al., 2001) or probably high RoB. Even more, in one of the 6 studies (Tzee‐Cheng et al., 1991), a co-intoxication occurred with excessive levels of boric acid (> 20 mg/dl in urine of cases versus healthy adults or children in another country), presumably added as a prohibited preservative to consumed rice noodles, which could have had an influence on the severity and outcome of the acute aflatoxicosis (Tzee‐Cheng et al., 1991). Three studies scored a probably low RoB, based upon the ideal aflatoxin measurement (*i.e.* AFB1 albumin or AFB1 lysine adduct in plasma or serum), and the consideration of other influencing factors such as exposure to other toxins, co-existence of virology factors (*e.g.* acute liver failure induced by hepatitis B), as well as liver failure due to medication and/or alcohol use. Confidence of exposure characterization (**Question 2**) varied according to the method for diagnosis, with studies where aflatoxins were measured in plasma, serum and tissue extracts (n = 4) resulting in definitely low RoB, studies in which aflatoxins were measured in consumed food (n = 1) (Samuel, 2009) or food from affected areas (n = 2) (Nyikal et al., 2004, Perduri and Gobba, 2009) resulting in probably low RoB, and studies without any information on aflatoxin measurement resulting in probably high RoB (n = 1) (Ombui et al., 2001). Furthermore, acute aflatoxicosis was confidently diagnosed in a hospital setting (**Question 3**) in 5 out of 9 studies, resulting in a definitely low RoB. For 2 other studies, although in a hospital setting, diagnosis was based on exclusion and consumption of AF-contaminated food and/or similar symptoms in in-house animals, resulting in a probably high RoB (Perduri and Gobba, 2009, Samuel, 2009). For one study, no information was provided about the diagnosis method, again resulting in a probably high RoB (Ombui et al., 2001). Finally, during outbreaks, and inherent to outbreak reports, the omission of less severe cases (**Question 4**) (*i.e.* not admitted to health facilities or hospitals) leads to a probably high RoB (n = 5). Individual case reports were assessed to be at low RoB (n = 2) (Perduri and Gobba, 2009, Samuel, 2009), whilst 1 study in which only volunteers were included (Jolly et al. 2007), was designated as having a definitely high RoB.

Based upon the results from each question, 5 studies were assessed to be at low RoB. This means that these studies were considered well-designed, conducted, and analyzed, and thus, their results were likely reliable and trustworthy. Three studies were assessed to be at uncertain RoB, raising concerns on some aspects of these studies (*e.g.* lack of proper confounding, variation in aflatoxin analysis method, omission of less severe cases, or co-intoxication with other food contaminant) making it unclear whether bias might have influenced the results. Only one study (Ombui et al. 2001) was assessed to be at high RoB, implying significant flaws in its study design, conduct, and/or analysis which could have introduced bias that might have compromised the validity of its findings. However, because this study offered limited information − lacking population sample size for incidence calculations, mortality data, and details on symptom duration and type − the high RoB was not anticipated to significantly impact the final evidence synthesis of this SR.

#### 3.4.2 Certainty of body of evidence

The overview of the GRADE assessment, including the rationale of decisions, has been detailed in Table 7. Initially this review was rated as “low” due to all observational study designs, including cross-sectional studies and outbreak/case reports. It was further downgraded for **inconsistency**: studies were conducted in small areas across six different countries and, although all defined as aflatoxicosis, the exposure assessment methods to diagnose the disease varied widely. Additionally, one study based its diagnosis on the combined exposure to boric acid and aflatoxins (Tzee‐Cheng et al., 1991). However, it was not downgraded for **indirectness**, as the studied populations showed minimal differences, covering all ages, originating primarily from low-income countries, and shared a consistent focus on aflatoxin exposure, with similar outcomes.

**Table 7:** Detailed overview, including the criteria & rationale for decisions of the GRADE assessment of the overall body of evidence of this SR (Morgan et al., 2016).

	**CRITERIA**	**Answer**	**Rationale**
**Initial certainty based on study design**	Randomized controlled trials = high. Observational studies = low.	Low (grade value)	Cross-sectional studies, and case and outbreak reports.
**Downgrade: Inconsistency**	1. Are there different subgroups of patients with different effects? 2. Was the outcome differently defined between studies? 3. Was the conduct of studies with varying quality?	Yes (−1)	√ Yes, small localized areas in 6 different countries. √ Yes, the diagnosis varied − some studies used food (or feed) analysis, others measured AFs in serum. One study only “tentatively” identified cases with minimal information, while another could not distinguish between boric acid and aflatoxin exposure. √ Yes, RoB ranged between high, low and uncertain.
**Downgrade: Indirectness**	Are there differences in: 1. populations? 2. exposure? 3. outcome?	No (0)	X No, little differences in studied populations: all ages, all low-income countries. X No, all high aflatoxin exposure. X No, all studied outcomes are related to acute aflatoxicosis.
**Downgrade: Imprecision**	Is there an inadequate sample size (suboptimal information size, no sample size calculation, sample sizes < 100)?	Yes (−1)	√ Yes, only one study provided the population sample size able to calculate incidence, and the number of cases reported in most studies was generally low (≤ 68 cases, except for one study which reported 317 cases). None of the studies reported a sample size calculation.
**Downgrade: Publication bias**	Are results prone to bias due to file-drawering effect?	Yes (−1)	√ Yes, milder cases, despite potentially high AF exposure, were likely underrepresented as they were not admitted to the hospital and thus unrecorded. This underreporting was exacerbated by hospital admission biases, favoring individuals with the financial means, typical in outbreak settings in low-income countries.
**Upgrade: magnitude of effect**	Upgrade with 1 level if risk reduction ± 50 % or OR ≥ 2? Upgrade with 2 levels if risk reduction ± 80 % or OR between 5 and 10?	Yes (+1)	√ Yes, calculated odds ratios indicated a higher risk of acute aflatoxicosis symptoms with values ranging from 2.2 to 5.1, at elevated aflatoxin levels (≥250 pg/mg albumin in serum).
**Upgrade: presence of a clear dose–response gradient**	Presence of a clear dose–response gradient?	No (0)	X No, symptoms of aflatoxicosis were observed across a wide range of AF concentrations: 10 to 51,100 µg/kg in food, 36 to 209,000 pg/mg albumin in serum, and 19 to 18,521 pg/g in tissue.
**Upgrade: accounting for residual confounding**	Did studies account for (residual) confounding?	No (0)	X No, except for one study, no studies controlled for confounders.
**Overall GRADE**	High: very confident that true effect lies close to that of estimate effect. Moderate: moderately confident that true effect lies close to the effect estimate. Low: Confidence in the effect estimate is limited. Very low: very little confidence in the effect estimate.	Very low (Sum = -2)	Based on the inconsistency and imprecision between studies, as well as the risk for publication bias, lack of matching for confounding and a clear dose–response gradient, the overall quality from the SR was determined to be very low.

Note: AF = aflatoxin; RoB = Risk of bias.

A further downgrade was deemed appropriate for **imprecision**: only one study provided population sample sizes to calculate incidence, and the number of cases reported in most studies was generally low (≤68 cases, except for Nyikal’s study (Nyikal et al., 2004), which reported 317 cases). Lastly, another downgrade was applied for **publication bias**, as less severe cases − despite potentially high AFB1 consumption − were likely underrepresented, *i.e.* not admitted to the hospital and thus not recorded. This underreporting was further compounded by the probability of hospital admission biases, where only individuals financially capable of admission are typically included in an outbreak setting in a low-income country (Kitano et al., 2021).

Only one study (Jolly et al., 2007) provided odds ratios indicating a higher risk of symptoms such as painful vomiting, swollen stomach, and yellow mouth, with values ranging from 2.2 to 5.1 at elevated aflatoxin levels (≥250 pg/mg albumin in serum). An uprating based on the **magnitude of effect** was considered acceptable for this finding. However, no uprating was granted for a **clear dose**–**response gradient**, as symptoms of aflatoxicosis were observed across a wide range of aflatoxin concentrations. Lastly, except for one study (Kinyenje et al., 2023), no studies **controlled for confounders**, and thus no uprating was applied for this factor. After accounting for all factors influencing the upgrading and downgrading of evidence, the overall quality from the SR was determined to be very low. This means that upon today, there is insufficient evidence available to assess the true burden of acute aflatoxicosis, and highlights the critical need for a standardized and well-structured reporting guideline to enhance evidence quality. Nevertheless, the evidence remains valuable, as each reported case of acute aflatoxicosis can provide essential data for calculating incidence and mortality rates, as well as inform on disease duration, and symptomatology.

## 4 Discussion

**Paucity of epidemiological studies** − The small number of reported studies with varying quality, as well as the observed heterogeneity in study design, geographic distribution, age group, and aflatoxin type and matrix analysis, underlines the paucity of epidemiological studies on acute aflatoxicosis. Overall, no aflatoxicosis-related population cohort has been conducted, and reports have not been documented in America and Australia. Furthermore, the ability to directly compare aflatoxin exposure levels is complicated by the use of different matrices across studies. When focusing on biological assessments, various biomarkers are used, including AFB1 albumin adducts (Jolly et al., 2007, Kamala et al., 2018), its digestion product AFB1 lysine adduct (Kinyenje et al., 2023), or the aflatoxin parent compounds (Mwanda et al., 2005; Tzee‐Cheng et al., 1991), each presenting unique challenges. One issue is their differing half-lives: AFB1 albumin or lysine adducts have a relatively long half-life of 2 to 3 months in blood, offering a marker for cumulative exposure over extended periods (Li et al., 2019). The AFB1 parent compound, being rapidly metabolized and cleared from the bloodstream, indicates immediate exposure, potentially underestimating long-term exposure relative to adducts (Autrup and Autrup, 1992). Another challenge involves the sensitivity of the detection methods – if reported –, which varies depending on the matrix and the employed methodology, although variation can also exist within similar methodologies due to difference in sample preparation, extraction techniques, instrument settings, reagent quality, and/or operator practices (Mahfuz et al., 2020). The variability in detection limits and sensitivities across these methods can lead to either under- or overestimation of exposure, further complicating comparisons between biomarkers. Considering all factors affecting the evaluation of evidence quality, it was found that current evidence is insufficient to accurately determine the true burden of acute aflatoxicosis although each documented case provided valuable insights into the incidence, mortality and symptomology/disease duration of acute aflatoxicosis.

**Absence of uniform reporting guideline** − Within the scarcely reported outbreak or cross-sectional studies, often crucial data such as population size, which is essential in calculating the incidence, as well as mortality numbers, and duration of disease/pathology are missing, implying the need for a well-structured, uniform, reporting guideline. In this guideline, standardized aflatoxin measurement techniques should be prioritized, along with a thorough consideration of confounding factors, as highlighted by the RoB quality assessment, where six out of nine studies scored poorly in this area. Guidelines should, at a minimum, include reporting of population sizes, incidence data, biological aflatoxin measurements, a comprehensive and long-term follow-up with documentation of symptomology data, disease duration and the occurrence of mortality, ideally collected from hospital records. To date, there are no established templates for the minimal reporting of acute aflatoxicosis outbreaks, as per the EQUATOR (Enhancing the QUAlity and Transparency Of health Research) Network (https://www.equator-network.org/) (Simera et al., 2010). It is imperative to initiate efforts, coordinated by WHO, to create such a reporting template which should be referenced to in the World Health Organization Outbreak Communication Planning Guide (WHO, 2008a) and the Guideline for investigation and control of foodborne disease outbreaks (WHO, 2008b). To this end, the Global Environment Monitoring System (GEMS) could support WHO's efforts to develop a minimal reporting template for acute aflatoxicosis outbreaks by integrating environmental monitoring data including crop contamination data, temperature, humidity and other factors that influence fungal growth and toxin production, fostering standardization, and building local capacities, all of which are essential for effective outbreak communication and response (Mesfin et al., 2021).

**Lack of reference values** − Although aflatoxin food concentrations associated with acute aflatoxicosis are slightly lower, equal to, but most often exceed the maximum levels set by the European Commission (between 4 and 10 µg/kg for various foodstuffs), no definite conclusion can be drawn to which concentration in serum, plasma, tissue, food or feed is associated with disease onset based on the broad concentration ranges reported within this review, despite that more cases seem to occur in children less than 15 years of age (Nyikal et al., 2004), probably because of their larger intake per body weight ratio and their lower capacity to detoxify (Andrews-Trevino et al., 2021). Currently, there are no globally established reference values or human biomonitoring (HBM) guidelines specifically for aflatoxin levels in blood. Regulatory agencies, such as the European Commission focus primarily on setting limits for aflatoxins in food and agricultural products to control exposure, rather than establishing blood concentration reference values, although research has shown plasma concentrations as low as 5 pg/mg albumin being associated with an increased risk of hepatocellular cancer (Chen et al., 2009). Next to that, assessing exposure from the consumption of meat produced by animals fed high-aflatoxin diets, as in the study of Perduri and Gobba (2009), is complex. Research indicates that in ruminants, aflatoxins are partially detoxified in the rumen, allowing for only 40 % of the toxins to reach muscle tissues, *i.e.* a repetitive exposure of 4.0 µg/kg body weight (BW) results in muscle concentrations of 1.6 µg/kg, although only assessed in heart muscle tissue (Guo et al., 2021). When implementing this to the case report of Perduri and Gobba (2009) (Perduri and Gobba, 2009), and assuming an adult heifer weighs 500 kg and consumes 10 kg of feed daily (Krizsan et al., 2014), the resulting exposure would be 0.5 µg/kg of BW, leading to 0.2 µg/kg of AFB1 potentially accumulating in meat. This AFB1 concentration is well below the maximum allowable levels for any foodstuffs, although no maximum levels have yet been set for AFB1 in meat itself (European Commission, 2023). In contrast, studies show that almost 95 % of AFB1 consumed by dairy cows is metabolized into AFM1, and excreted as such in milk. Applying this to the study of Perduri and Gobba (2009) (Perduri and Gobba, 2009), a concentration level of approximately 0.5 µg/L in milk exceeds the European limit by tenfold for both raw and heat-treated milk being 0.05 µg/L. Although AFM1 is hepatotoxic like AFB1, it is however less potent than AFB1 and primarily associated with chronic health effects rather than acute aflatoxicosis (Gao et al., 2022).

**Golden standard** − To get a better insight into the toxicological profiles, future endeavors should focus on diagnosing acute aflatoxicosis based on a combination of clinical presentation, history and direct analysis of aflatoxin biomarkers, preferably the ones for cumulative exposure, in serum and/or plasma by accurate and precise methodologies such as LC/MS-MS. To that end, AFB1 lysine standards should be made commercially available, as their current unavailability hinders quality research, and although these are essential for studies on aflatoxin exposure, the niche demand discourages large-scale production (Smith et al., 2022). Additionally, to prevent the omission of milder acute aflatoxicosis cases, which often goes unnoticed in outbreak reports, raising awareness at both global and local levels is crucial for improving disease reporting and more accurately estimating the true burden of acute aflatoxicosis. To this end, several strategies can be employed, such as the implementation of health campaigns in aflatoxin-prone regions, training of local healthcare workers, strengthening of regional laboratories by international collaborations and the leverage of international organizations like WHO to promote global awareness as has been done by means of the current SR in the pursuit of systematic data collection on acute aflatoxicosis.

**Need for molecular epidemiology** − Finally, although outbreak reports and traditional epidemiological studies help establish correlations between aflatoxin exposure and health outcome, offering a broad understanding, they lack the precision needed to fully explain biological pathways linking aflatoxin exposure to acute toxicity. Molecular epidemiology could provide more robust evidence that directly links aflatoxin exposure to biological changes at the molecular and cellular levels, focusing on molecular markers, such as aflatoxin-protein adducts, aflatoxin-phospholipid adducts, aflatoxin-DNA adducts gene expression changes, or biomarkers of oxidative stress and inflammation (Benkerroum, 2020). This kind of evidence is particularly important for acute aflatoxicosis because of its rapid onset and severe nature, which suggests a direct and potent biological effect, possibly through pathways involving liver enzymes, protein & phospholipid damage, and immune system disruption. However, similar to the scarcity of epidemiological studies, there is also a lack of molecular epidemiology research in the field of acute aflatoxicosis, which limits our understanding of how aflatoxins cause acute toxic effects in humans (Benkerroum, 2020). To address this, standardized epidemiological studies should be combined with molecular approaches. In outbreak settings, for example, biological samples (*e.g.* blood, urine, liver biopt tissue) from affected individuals could be analyzed not only for aflatoxin concentrations but also for molecular biomarkers, which offer insights into the biological pathways underlying acute aflatoxicosis. By incorporating standardized molecular tests into a standardized outbreak reporting guideline, more comprehensive and mechanistic data that complement traditional epidemiological findings can be obtained.

**Strengths and limitations of this SR** − A key strength of this SR is its comprehensive nature, which encompasses diverse data sources, multiple countries, and a multidisciplinary team of leading international experts. This approach ensures a robust search strategy across relevant databases and the inclusion of the most pertinent grey literature. By examining various aflatoxin detection methods and biomarkers, this review acknowledges differences in exposure assessment techniques, enabling a deeper understanding of their impact while also providing guidance for the standardization and harmonization of methods, data reporting, and study findings. A major limitation lies in the retrieval of a limited number of carried-out studies with overall high heterogeneity. The absence of data from regions like America and Australia further restricts generalizability. Variations in biomarkers, detection methods, and sensitivity levels complicate direct comparisons of aflatoxin exposure and associated health risks. Next to that, many studies fail to report crucial epidemiological data, such as population size, incidence rates, and mortality figures, making it difficult to assess the true impact of acute aflatoxicosis. Lastly, unreported or misdiagnosed cases, due to inadequate surveillance and healthcare infrastructure, likely lead to an underestimation of the disease burden, reinforcing the need for improved public health strategies and systematic outbreak reporting.

## 5 Conclusion

This SR assessed the available national or (sub-)regional evidence on incidence and mortality, as well as symptomology and disease duration for additional clinical context, of acute aflatoxicosis, from 1990 onwards. Based upon the in- and exclusion criteria, a total of 9 out of 11,539 references obtained from 5 major bibliographic databases and bibliographic screening were retained into the final report. Between studies, heterogeneity existed based on study design (case reports, outbreak reports and cross-sectional studies), geographic region (Africa, Asia, Middle-East and Europe; primarily sub-national level), age (between 0 and 86 years) and aflatoxin type & matrix analysis (either as albumin or lysine adducts, or as parent compound in biological samples, consumed food, and food and feed from affected areas with varying analytical techniques and sensitivity). Reported cases of acute aflatoxicosis ranged between 1 (case reports) and 317, however, the population sample size was reported in only one study, *i.e.* an outbreak report in the United Republic of Tanzania, covering 3 local districts, and resulting in a disease-attack rate of 8 cases per 100,000. Case-reported aflatoxin concentrations ranged between 10 and 51,100 µg/kg in food, 36 and 209,000 pg/mg albumin in serum, and 19 and 18,521 pg/g in tissue. Case fatality rates ranged between 16.2 and 76.5 % according to different outbreak studies. Highest mortality rates were found for age groups > 40 and ≤ 15 years of age, however taking into account widely overlapping confidence intervals. Overall reported symptoms included vomiting, jaundice/yellow mouth, and abdominal swelling/pain, with a disease duration between 1 and 48 days, except for a case with repetitive episodes over the course of 33 months. Out of 9 studies, 5 studies scored an overall low RoB based on potential confounding, confident outcome and exposure measurement, and selective reporting. Furthermore, the overall CoE assessment highlighted the lack of evidence available to assess the true burden of acute aflatoxicosis, although the disease remains a significant public health burden, especially among vulnerable groups in African countries. Next to that, the review stresses the paucity of epidemiological studies on acute aflatoxicosis, with varying quality, and a large heterogeneity in study design, geographic distribution, age group, and aflatoxin matrix analysis. Overall, the lack of data calls for more rigorous and standardized investigations with the establishment of a reporting guideline, to enhance our knowledge and inform effective prevention and intervention strategies, and enable disease burden estimation despite challenges with infrastructure and resources in affected areas.

## CRediT authorship contribution statement

**Tess Goessens:** Writing – original draft, Validation, Supervision, Software, Project administration, Methodology, Investigation, Formal analysis, Data curation, Conceptualization. **Kokeb Tesfamariam:** Writing – review & editing, Investigation, Formal analysis. **Patrick Berka Njobeh:** Conceptualization, Writing – review & editing, Funding acquisition. **Limbikani Matumba:** Conceptualization, Writing – review & editing, Funding acquisition. **Nyadani Jali-Meleke:** Validation, Investigation, Data curation. **Yun Yun Gong:** Conceptualization, Writing – review & editing, Funding acquisition. **Zdenko Herceg:** Writing – review & editing, Funding acquisition. **Chibundu N. Ezekiel:** Conceptualization, Writing – review & editing, Funding acquisition. **Sarah De Saeger:** Conceptualization, Writing – review & editing, Funding acquisition. **Carl Lachat:** Conceptualization, Writing – review & editing, Funding acquisition. **Marthe De Boevre:** Conceptualization, Validation, Supervision, Writing – review & editing, Project administration, Funding acquisition.

## Funding

This research was commissioned and funded by the World Health Organization (Reference No CTTF-001). Copyright in the original work in which this article is based belongs to WHO. The authors have been given permission to publish this article.

## Declaration of competing interest

The authors declare that they have no known competing financial interests or personal relationships that could have appeared to influence the work reported in this paper.

## Data Availability

We have shared the raw data via the open-access platform OSF as acknowledged into the manuscript (https://osf.io/zj9kp/)
