# Supplementary material for: Incidence and mortality of acute aflatoxicosis: A systematic review
Source: Environ Int. 2025 May;199:109461. doi: 10.1016/j.envint.2025.109461 (PMC12086173; doi:10.1016/j.envint.2025.109461)
Supplement: Supplementary Data 1 [file mmc1.docx]

**Incidence and mortality of acute aflatoxicosis:**

**a systematic review.**

***Tables***

**Table S1:** detailed information regarding the review team, advisory panel and sponsor involved in this systematic review.

**Table S2:** detailed search strings for each of the search engines included within the SR.

**Table S3.** Detailed overview, including the rationale for decisions, of RoB assessment of the eligible studies included in this SR.

**Table S1:** detailed information regarding the review team, advisory panel and sponsor involved in this systematic review.

| ***Review team*** |
| --- |
| A multidisciplinary team consisting of scientists from Ghent University (Belgium, Europe), University of Johannesburg (South Africa, Africa), Lilongwe University of Agriculture and Natural Resources (Malawi, Africa), University of Leeds (England, United Kingdom), University of Natural Resources and Life Sciences Vienna (Austria, Europe), and the International Agency for Research on Cancer (IARC) (WHO, Lyon), with particular expertise in mycotoxicology, human health, nutrition and SR methodologies, performed the study. Each author was assigned a specific role based on their expertise (See Table below). TG has been the guarantor of the SR, responsible for the overall scientific integrity of the work and for developing and implementing an effective communication and project management strategy. TG has been involved in coordinating and integrating input from all experts on the research team throughout the various stages of the SR. For optimal project communication, management strategy and progress evaluation, monthly meetings were organized to aid in the preparation of the review protocol, research questions, search strings tailored to each search engine, study selection, quality of evidence, evidence synthesis and points of discussion.   \| **Assigned role** \| **Author** \| \| --- \| --- \| \| Scoping exercise for input search strategy,  database search & protocol development \| TG, CL, MDB, PN, YYG, CNE, LM \| \| Screening of title, abstract & full-text \| TG, NJ, CL \| \| Data extraction \| TG, NJ \| \| Quality assessment \| TG, MDB, CL* \| \| Evidence synthesis \| TG, KT, CL \| \| Report writing \| TG, CL, MDB, SDS, PN, LM, CNE, YYG, ZH \|   Note: TG : Tess Goessens, CL = Carl Lachat, MDB = Marthe De Boevre, PN = Patrick Njobeh, YYG = Yun Yun Gong, NJ = Nyadani Jali, CNE = Chibundu N. Ezekiel, LM = Limbikani Matumba, KT = Kokeb Tesfamariam, SDS = Sarah De Saeger, ZH = Zdenko Herceg. *All authors have thoroughly reviewed the final assessment. |
| ***Advisory panel*** |
| An advisory panel was established to ensure broad stakeholder representation, incorporating experts in toxin and food safety science, as well as specialists in public health risk assessment and epidemiology. The panel consisted of five members: Professor Dr. Arie Hendrik Havelaar, a leading scientist in Global Food Safety and Zoonoses within the Animal Sciences Department, the Emerging Pathogens Institute, and the Food Systems Institute at the University of Florida, Gainesville, USA; Professor Dr. Brecht Devleesschauwer, Head of Service Health Information at Sciensano, Brussels, Belgium, and guest lecturer at the Department of Translational Physiology, Infectiology, and Public Health, Faculty of Veterinary Medicine at Ghent University, Merelbeke, Belgium; Dr. Antonio Agudo Trigueros, senior researcher at the Catalan Institute of Oncology (ICO) Unit of Nutrition, Environment, and Cancer, Barcelona, Spain, and part of the coordinating team for the "European Research in Cancer and Nutrition" (EPIC) multicenter study; Dr. Lea Sletting Jakobsen, senior researcher at the National Food Institute, Technical University of Denmark, Kgs Lyngby, Denmark; and Carlotta Di Bari, Health Information Scientist at Sciensano, Brussels, Belgium. This support was provided under the name of WHO’s FERG. Specifically, Dr. Lea Sletting Jakobsen and Dr. Antonio Agudo Trigueros, both FERG members, played key roles in assisting the team as co-chairs of the FERG Chemicals and Toxins Task Force, maintaining their objectivity and impartiality in their contributions. Professor Dr. Arie Havelaar provided his support as a WHO consultant, being compensated by WHO for his work. Additionally, the support from Professor Dr. Brecht Devleesschauwer and Carlotta Di Bari was also provided under WHO, as Sciensano was commissioned by WHO to monitor the progress of the data collection. The advisory group provided strategic input during the planning and scoping phases, assisting the research team in identifying relevant background information, framing the research question, constructing the research string, identifying pertinent grey literature and databases and constructing the protocol. Throughout the project, they continued to offer strategic support, including developing a data extraction template and contributing to the interpretation of findings. However, the advisory panel was not involved in the operational execution of the work. The review guarantor (TG) facilitated coordination with the advisory panel, ensuring clear and efficient communication between the review team and the advisors through monthly meetings and additional interactions as needed. |
| ***Sponsor*** |
| This research was commissioned and funded by the WHO. WHO provided advisory support at the project's outset, focusing on planning, orientation, project management, and prioritization. The sponsor had no involvement in the conduct of the work, including scoping, setting Population, Exposure, Comparator, and Outcome (PECO) elements, constructing the search string, developing the protocol, or writing the manuscript. The authors have been given permission by WHO to publish this article.  The sponsor was represented by Yuki Minato, technical officer at the department of Nutrition and Food safety of WHO, Geneva, Switzerland. |

**Table S2:** detailed search strings for each of the search engines included within the SR.

| EMBASE |
| --- |
| (afla*:ti,ab,kw OR mycotoxin*:ti,ab,kw OR ‘fungal toxin*’:ti,ab,kw OR ‘mycotoxin’/exp OR ‘aflatoxin’/exp OR ‘aflatoxin B’/exp OR ‘aflatoxin B1’/exp OR ‘aflatoxin B2’/exp OR ‘aflatoxin G1’/exp OR ‘aflatoxin G2’/exp OR ‘aflatoxin M1’/exp) AND (aflatoxicosis:ti,ab,kw OR ‘aflatoxin poisoning’:ti,ab,kw OR ‘aflatoxin toxicity’:ti,ab,kw OR ‘acute hepatic necrosis’:ti,ab,kw OR ‘acute liver failure’:ti,ab,kw OR ‘acute hepatotoxicity’:ti,ab,kw OR ‘aflatoxin-induced illness’:ti,ab,kw OR ‘aflatoxicosis’/exp OR ‘acute liver failure’/exp) NOT (‘animal’/exp not ‘human’/exp)  Filter: publications from 1990 - 2023 |
| PubMed |
| ((afla* OR mycotoxin* OR “fungal toxin*”) AND (aflatoxicosis OR “aflatoxin poisoning” OR “aflatoxin toxicity” OR “acute hepatic necrosis” OR “acute liver failure” OR “acute hepatotoxicity” OR “aflatoxin-induced illness”)) NOT (“animal” not “human”)  Filter: publications from 1990 - 2023 |
| Web of Science |
| ((afla* OR mycotoxin* OR “fungal toxin*”) AND (aflatoxicosis OR “aflatoxin poisoning” OR “aflatoxin toxicity” OR “acute hepatic necrosis” OR “acute liver failure” OR “acute hepatotoxicity” OR “aflatoxin-induced illness”)) NOT (“animal” not “human”)  Filter: publications from 1990 - 2023 |
| Scopus |
| TITLE-ABS-KEY (((afla* OR mycotoxin* OR “fungal toxin*”) AND (aflatoxicosis OR “aflatoxin poisoning” OR “aflatoxin toxicity” OR “acute hepatic necrosis” OR “acute liver failure” OR “acute hepatotoxicity” OR “aflatoxin-induced illness”)) NOT (animal not human))  Filter: publications from 1990 - 2023 |

**Table S3.** Detailed overview, including the methodology & rationale for decisions of the RoB assessment of the included eligible studies.

| **Source** | **1. Did the study design or analysis account for important confounding and modifying variables?** | **2. Can we be confident in the exposure characterization?** | **3. Can we be confident in the outcome measure?** | **4. Were all measured outcomes reported?** | **Overall RoB** |
| --- | --- | --- | --- | --- | --- |
| **CRITERIA** | - Definitely low if 3 criteria fulfilled: matching of groups (Truong et al., 2022), reliable measurement = in biological samples (Mahfuz et al., 2020), and taken into account at least one influencing factors such as viral, parasitic or bacterial infections (Kimani, 2022) - Probably low: 2 out of 3 fulfilled - Probably high: 1 out of 3 fulfilled - Definitely high: none fulfilled | - Definitely low: AFs measured in biological samples (Mahfuz et al., 2020) - Probably low: AFs measured in food (Mahfuz et al., 2020) - Probably high: very little info on the measurement - Definitely high: no info on measurement | - Definitely low: diagnosed in hospital - Probably low: diagnosed via trained personnel outside hospital setting or by exclusion within hospital setting - Probably high: diagnosed via questionnaire - Definitely high: no info on diagnosis | - Definitely low: direct proof that all outcomes were documented (*i.e.* hospital records) - Probably low: indirect proof that all outcomes were documented (*i.e.* medical records) - Probably high: indirect proof that not all outcomes have been documented (*e.g*. loss of follow-up, outbreak reports) - Definitely high: direct proof that not all outcomes have been documented (*e.g.* biased inclusion) | - Low: 3 out of 4 questions answered with low RoB Uncertain: equal amount of questions answered with low and high RoB - High: 3 out of 4 questions answered with high RoB |
| **(Jolly et al., 2007)** | Probably low RoB | Definitely low RoB | Definitely low RoB | Definitely high RoB | Low |
| ***Rationale*** | *Unmatched, reliable measurement √, taking into account other influencing factors (i.e. viral hepatitis) √* | *AFB1 measured in biological samples (i.e. serum)* | *Diagnosed in hospital setting* | *Only inclusion of volunteers* |  |
| **(Kamala et al., 2018)** | Probably low RoB | Definitely low RoB | Definitely low RoB | Probably high RoB | Low |
| ***Rationale*** | *Unmatched, reliable measurement √, influencing factors taken into account (i.e. bacterial infection, viral hepatitis & viral hemorrhagic fever) √* | *AFB1 albumin adduct measured in biological samples (i.e. serum)* | *Diagnosed in hospital setting* | *Medical record review & patient interviews, however inherent to outbreaks: less severe cases not admitted to hospital/health facility* |  |
| **(Kinyenje et al., 2023)** | Probably low RoB | Definitely low RoB | Definitely low RoB | Probably high RoB | Low |
| ***Rationale*** | *Unmatched, reliable measurement √, information regarding other influencing factors (i.e. chronic medications and chronic use of alcohol) √* | *AFB1 lysine measured in biological samples (i.e. serum)* | *Diagnosed in hospital setting* | *Medical record review & patient interviews, however inherent to outbreaks: less severe cases not admitted to hospital/health facility* |  |
| **(Mwanda et al., 2005)** | Probably high RoB | Definitely low RoB | Definitely low RoB | Definitely low RoB | Low |
| ***Rationale*** | *Unmatched, reliable measurement √, not taking into account other influencing factors* | *AFs measured in biological samples (i.e. serum)* | *Diagnosed in hospital setting* | *Hospital records of all cases* |  |
| **(Nyikal et al., 2004)** | Probably high RoB | Probably low RoB | Definitely low RoB | Probably high RoB | Uncertain |
| ***Rationale*** | *Matched √, aflatoxins measurement in food from affected areas, not taking into account other influencing factors* | *Aflatoxins measured in food from affected areas (i.e. in-house maize flour, maize grains, dry maize cobs, muthokoi, millet, sorghum, and beans)* | *Diagnosed in hospital setting* | *Medical record review & patient interviews, however discontinuation of follow-up in 7 health centres + inherent to outbreaks: less severe cases not admitted to health facility* |  |
| **(Ombui et al., 2001)** | Definitely high RoB | Probably high RoB | Probably high RoB | Probably high RoB | High |
| ***Rationale*** | *Unmatched, no detail about measurement and only in food, no recognition influencing factors - tentatively diagnosed* | *Very little info provided about measurement: tentatively diagnosed based on food analysis but not specified which foods or at which concentrations* | *Insensitive diagnosis method – questionnaire* | *Retrospective annual reports of outbreaks - inherent to outbreaks: less severe cases not reported* |  |
| **(Perduri and Gobba, 2009)** | Probably high RoB | Probably high RoB | Probably low RoB | Definitely low RoB | Uncertain |
| ***Rationale*** | *Unmatched, unreliable measurement in feed, taking into account other influencing factors (i.e. viral hepatitis, microorganisms, parasites, celiac disease, pancreatitis) √* | *Aflatoxins measured in feed from presumably consummated animals/meat/milk* | *Diagnosed based on exclusion by repeated medical examinations and high symptoms of aflatoxicosis in farm animals from which the person was consuming meat/milk* | *All-reporting hospital record* |  |
| **(Samuel et al., 2009)** | Probably high RoB | Probably low RoB | Probably low RoB | Definitely low RoB | Low |
| ***Rationale*** | *Unmatched, unreliable measurement in canned food, taking into account influencing factors (i.e. intoxication with methanol, ethylene glycol and acetyl salicylic acid, as well as virology, bacteriology and parasites) √* | *Aflatoxins measured in consumed canned food** | *Diagnosed based on exclusion within hospital setting and high AFB1 levels in consumed canned food* | *All-reporting hospital record* |  |
| **(Tzee‐Cheng et al., 1991)** | Probably high RoB | Definitely low RoB | Definitely low RoB | Probably high RoB | Uncertain |
| ***Rationale*** | *Unmatched, reliable measurement √, the influencing factor of the boric acid has been acknowledged however effects cannot be distinguished from acute aflatoxicosis* | *Aflatoxins measured in post-mortem tissue (i.e. liver, lung, kidney, heart, brain, spleen)* | *Diagnosed in hospital setting* | *Only hospitalized cases, however inherent to outbreaks: less severe cases not admitted to hospital/health facility* |  |


**References**

Jolly, P.E., Jiang, Y., Ellis, W.O., Awuah, R.T., Appawu, J., Nnedu, O., Stiles, J.K., Wang, J.S., Adjei, O., Jolly, C.M., Williams, J.H., 2007. Association between aflatoxin exposure and health characteristics, liver function, hepatitis and malaria infections in Ghanaians. J. Nutr. Environ. Med. 16, 242–257. https://doi.org/10.1080/13590840701703918

Kamala, A., Shirima, C., Jani, B., Bakari, M., Sillo, H., Rusibamayila, N., De Saeger, S., Kimanya, M., Gong, Y.Y., Simba, A., Wigenge, R., Justin, I., Kyombo, F., Tarimo, V., Hipolite, D., Mziray, R., Kaiz, K., Mutabuzi, C., Muita, M., Mghamba, J., Mohamed, M.A., Kitambi, M., Nyanga, A., De Boevre, M., Xu, Y., Routledge, M.N., Mtui, N., Mosha, F., Charles, J., Manase, G., Lyamuya, F., Ngass, O.P., Ikaji, Z., Lyimo, C., Mchwampaka, W., Masumbuko, E., Magodi, R., Abade, A., Josephat, C., Omolo, J., Ruheta, M., Mengele, I., 2018. Outbreak of an acute aflatoxicosis in Tanzania during 2016. World Mycotoxin J. 11, 311–320. https://doi.org/10.3920/WMJ2018.2344

Kimani, P.M., 2022. Factors associated with prevalence of liver disease among seropositive HBV and AFB1 individuals in selected health facilities in Kitui and Makueni Counties, Kenya. Jomo Kenyatta University of Agriculture and Technology.

Kinyenje, E., Kishimba, R., Mohamed, M., Mwafulango, A., Eliakimu, E., Kwesigabo, G., 2023. Aflatoxicosis outbreak and its associated factors in Kiteto, Chemba and Kondoa Districts, Tanzania. PLOS Glob. Public Heal. 3, e0002191. https://doi.org/10.1371/journal.pgph.0002191

Mahfuz, M., Gazi, A., Hossain, M., Islam, M.R., Fahim, S.M., Ahmed, T., 2020. General and advanced methods for the detection and measurement of aflatoxins and aflatoxin metabolites: a review. Toxin Rev. 39, 123–137.

Mwanda, O.W., Otieno, C.F., Omonge, E., 2005. Acute aflatoxicosis: case report. East Afr. Med. J. 82, 320–324.

Nyikal et al., 2004. Outbreak of Aflatoxin Poisoning --- Eastern and Central Provinces, Kenya, January--July 2004. Morb. Mortal. Wkly. Rep. 53, 790–793.

Ombui, J.N., Kagiko, M.M., Arimi, S.M., 2001. Foodborne diseases in Kenya. East Afr. Med. J. https://doi.org/10.4314/eamj.v78i1.9111

Perduri, R., Gobba, S., 2009. Unexpected aflatoxin exposure in a woman in northern Italy: A case report. Cases J. 2. https://doi.org/10.4076/1757-1626-2-7736

Samuel, N., Ezri, Y., Farah, R., Igor, V., Hussein, A., Rubinshtein, O., Assy, N., 2009. Acute Aflatoxicosis Resulting in Fulminant Hepatic Failure and Rhabdomyolysis. Gastroenterol. Res 2, 48–50. https://doi.org/10.4021/gr2009.01.1254

Truong, N.N., Hadush, K.T., Visintin, L., Goessens, T., Saeger, S.D., Lachat, C., De Boevre, M.D., 2022. Associating Multiple Mycotoxin Exposure and Health Outcomes: Current Statistical Approaches and Challenges. World Mycotoxin J. 1–8.

Tzee‐Cheng, C., Maxwell, S.M., Wong, S. ‐Y, 1991. An outbreak of aflatoxicosis and boric acid poisoning in Malaysia: A clinicopathological study. J. Pathol. 164, 225–233. https://doi.org/10.1002/path.1711640307
